# The Macroscope: A tool for examining the historical structure of language

**DOI:** 10.3758/s13428-018-1177-6

**Published:** 2019-02-11

**Authors:** Ying Li, Tomas Engelthaler, Cynthia S. Q. Siew, Thomas T. Hills

**Affiliations:** 1 Department of Psychology, University of Warwick, Coventry, UK; 2 National University of Singapore, Singapore, Singapore

**Keywords:** Semantics, Language evolution, Language statistics, Word embedding

## Abstract

The recent rise in digitized historical text has made it possible to quantitatively study our psychological past. This involves understanding changes in what words meant, how words were used, and how these changes may have responded to changes in the environment, such as in healthcare, wealth disparity, and war. Here we make available a tool, the Macroscope, for studying historical changes in language over the last two centuries. The Macroscope uses over 155 billion words of historical text, which will grow as we include new historical corpora, and derives word properties from frequency-of-usage and co-occurrence patterns over time. Using co-occurrence patterns, the Macroscope can track changes in semantics, allowing researchers to identify semantically stable and unstable words in historical text and providing quantitative information about changes in a word’s valence, arousal, and concreteness, as well as information about new properties, such as semantic drift. The Macroscope provides information about both the local and global properties of words, as well as information about how these properties change over time, allowing researchers to visualize and download data in order to make inferences about historical psychology. Although quantitative historical psychology represents a largely new field of study, we see this work as complementing a wealth of other historical investigations, offering new insights and new approaches to understanding existing theory. The Macroscope is available online at http://www.macroscope.tech.

Hartley (1953) once wrote that “The past is a foreign country: They do things differently there.” Understanding why they did those things and what they were thinking when they did them is partly about history, but it is also falls under the umbrella of historical psychology. A number of recent accounts have documented apparent historical changes in the way people thought in the past. These accounts follow in the footsteps of well-documented historical changes that have taken place even in the last several centuries—for example, in the diffusion of print materials and the Industrial Revolution’s disarming of the Malthusian trap, releasing large parts of the world’s population from hand-to-mouth economies (Clark, 2008; Eisenstein, 1980). These changes have led to numerous claims explaining the rising spectre of risk in society (Beck, 1992), the whittling away of violent behavior by the civilizing process (Pinker, 2011), urbanization’s empowering of individuality and materialism (Greenfield, 2013), and the evolution of American English in response to information crowding (Hills & Adelman, 2015). The growing consensus appears to be that historical data represent a fertile ground for rolling our contemporary understanding of psychology back into the past.

The most common approach to studying historical beliefs and attitudes is what historians and literary critics call *close reading*. A close read involves a human reader, who reads over original texts, attending to individual words and sentences. Scaling this approach to the volume of historical text currently available, in order to make broad quantitative generalizations at the scale of hundreds of years, is effectively impossible. A person reading 50,000 words a day would require 22,000 years to close-read the text currently available in Google Ngrams book corpus. Over the past several decades, however, cognitive and language scientists have developed computational tools for *distant reading*, in which researchers use algorithms to extract meaning from billions of words of text. These have been used to study properties of word recognition (Jones & Mewhort, 2007), the structure of memory (Hills, Jones, & Todd, 2012), the relationship between natural language production and individual differences (Pennebaker & Stone, 2003), changing frequencies of word usage across individual lifespans (Le, Lancashire, Hirst, & Jokel, 2011), and changes in word use over hundreds of years (Michel et al., 2011). In doing so, this progression has moved language analysis from synchronic investigation of single words to diachronic investigations of texts across cultural time, all of which can take place within the lifetime of a single researcher (or even in an afternoon).

The goal of the present work is to introduce a tool that adds a further layer of structural depth to quantitative historical analysis, allowing researchers to zoom in and out on words—specifically, their semantics and the associations they maintained in historical language. We call this tool the *Macroscope*, after the device in Piers Anthony’s (1974) book of the same name, which could zoom in and out on the cultural history of alien civilizations. The key conceptual assumptions upon which the Macroscope stands are that words provide information about the past and that we can infer the meanings of words through the relations they keep with other words (e.g., Firth, 1957). Thus, meaning is derived through historical context, providing a new way of looking at semantic history. In what follows, we describe the underlying computational machinery of the Macroscope and provide several case studies that demonstrate the Macroscope’s utility for understanding historical language.

## Method

### The Macroscope

The Macroscope is a user interface consisting of a client–server interaction. The server, built in Node.js, handles user queries and analyses them in real time using Python. The data are then visualized on the client’s website. This tool can be found at http://www.macroscope.tech.

**Fig. 1:**
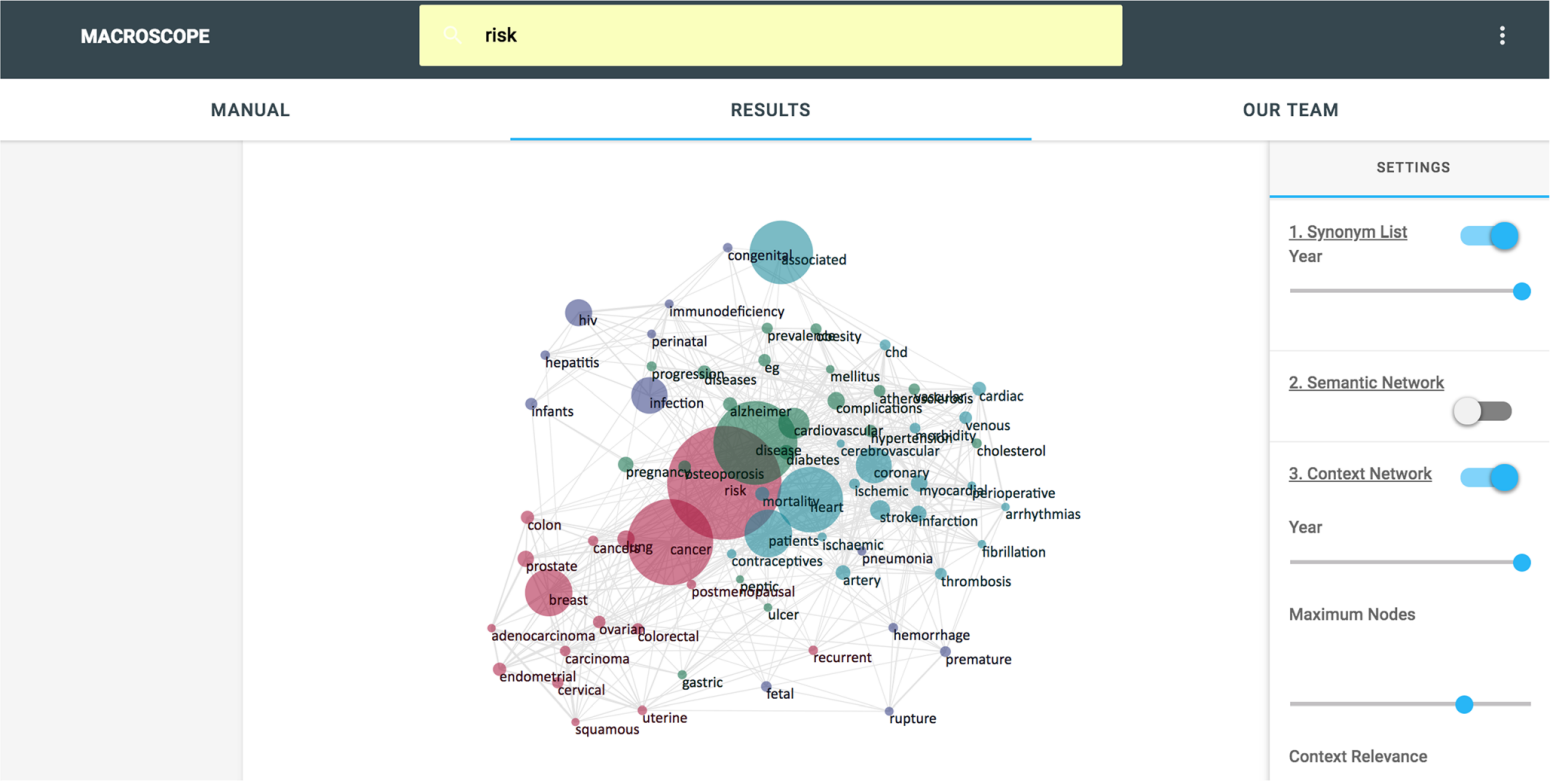
Screenshot of the Macroscope website. The search bar is at the top, where users can input the word of interest (*state* in the figure). The control panel on the right allows for selecting specific analysis and manipulating parameters

The Macroscope takes as input specific words of interest from the user, examines these in relation to a language corpus provided by the Macroscope, and outputs a range of historical indicators about changing semantics over time. Here we take semantics in the broadest possible sense (see below). Data for each historical indicator can be downloaded in .csv format to the user’s computer. A representation of the online interface for the Macroscope is shown in Fig. 1.

The details of the language corpora and computational algorithms are provided below.

### The language corpora

The current (first) iteration of the Macroscope uses text from the English Google Ngram Book corpus (specifically, 5-grams; Michel et al., 2011). This will be supplemented with additional corpora in forthcoming iterations, allowing users to compare data across multiple corpora. The Google Ngram Book corpus represents ~ 4% of all books published over the last several hundred years (Michel et al., 2011). Because the data representation is fairly sparse prior to 1800, we present data from 1800 to 2009, which contain approximately 155 billion words.

### Frequency

Usage frequency is computed by dividing the number of instances of a word in a given year by the total number of words in the corpus in that year. For instance, in 1861 the word *slavery* appeared in the corpus 21,460 times, on 11,687 pages of 1,208 books. The corpus contains 386,434,758 words from 1861; thus, the usage frequency of *slavery* in 1861 is 5.5 × 10^–5^. Users can input a search term into the search field and adjust various settings to capture and visualize the data of interest.

### Co-occurrence matrix

To compute word properties from the words that a given word co-occurs with, the Macroscope relies on co-occurrence. The Google Ngram data consist of a matrix using 5-gram data. The matrix records the number of times any two words co-occurred within a 5-gram over 209 years from 1800 to 2009. We include the top 50,000 most frequently used words across the 209 years, resulting in a 50,000 × 50,000 × 209 matrix. Each word in the co-occurrence matrix is represented as a vector of dimension 50,000 that stores its contextual information.

### Sentiment and concreteness

Using the co-occurrence matrix, the Macroscope computes contextual sentiment (valence), arousal, and concreteness by taking the mean of the relevant ratings of all the words that co-occurred with a given word in a given year. We used the Warriner, Kuperman, and Brysbaert’s (2013) norms to retrieve contemporary valence and arousal ratings for each word, and the Brysbaert, Warriner, and Kuperman (2014) norms to retrieve contemporary concreteness ratings for each word.

### Diachronic word embeddings

To find out which words are most semantically similar to each other and to quantify their degree of similarity, we used distributional semantics, in which words are embedded in vector space according to their co-occurrence relationships (Bullinaria & Levy, 2007; Turney & Pantel, 2010). We constructed diachronic word embeddings for each year in order to allow comparisons across different years. This approach has been effectively demonstrated in a number of studies (Hamilton, Leskovec, & Jurafsky, 2016; Sagi, Kaufmann, & Clark, 2011; Xu & Kemp, 2015)**.** In our study, we constructed word embeddings as follows. First, vectors containing the number of times a given word co-occurred with all other words were directly obtained from the co-occurrence matrix described above. Second, we computed the positive pointwise mutual information (PPMI) for each pair of words and then constructed a PPMI matrix with entries given by

$$ \mathrm{PPMI}\left({v}_i,{v}_j\right)=\max \left(0,\mathit{\log}\left(\frac{P\left({v}_i,{v}_j\right)}{P\left({v}_i\right)\times P\left({v}_j\kern0.1em \right)}\right)\right) $$

where *v*_*i*_, *v*_*j*_ represents a pair of words from the corpus, and *P*(*v*) corresponds to the empirical probabilities of word co-occurrences within a sliding window size of 5 over the original text. As compared to a simple co-occurrence count, PPMI penalizes high-frequency words (i.e., *of*, *the*, *and*) that are used in the same context with a wide range of words, and favors words that frequently appear together but not with others (i.e., *hong* and *kong*). Forcing PPMI values to be above zero ensures that they remain finite, and this has been shown to improve results (Bullinaria & Levy, 2007; O. Levy, Goldberg, & Dagan, 2015). Finally, we reduced the dimension of word embeddings to 300 using singular value decomposition (SVD). This dimensionality reduction acts as a form of regularization and allows us to compare word similarities by computing the cosine similarity of word embeddings.

To validate that the word embeddings we trained on the Google Ngram corpus accurately capture semantic relationships among the words, we tested these embeddings on 200 multiple-choice synonym questions collected by Levy, Bullinaria, and McCormick (2017). Each question corresponds to a set of five words: the test word, followed by the correct synonym, followed by three incorrect choices. Because some of the low-frequency words (such as *consommé* and *treacle*) were not included in our analysis, we tested 183 synonym questions using word embeddings trained on aggregated data from 2000 to 2008. Our performance (89.5% correct) was comparable to that of word embeddings trained using five different algorithms by Levy and his colleagues (accuracy rates ranging from 86.5% to 92.0%).

## Results

### Quantifying semantic and contextual change

**Fig. 2:**
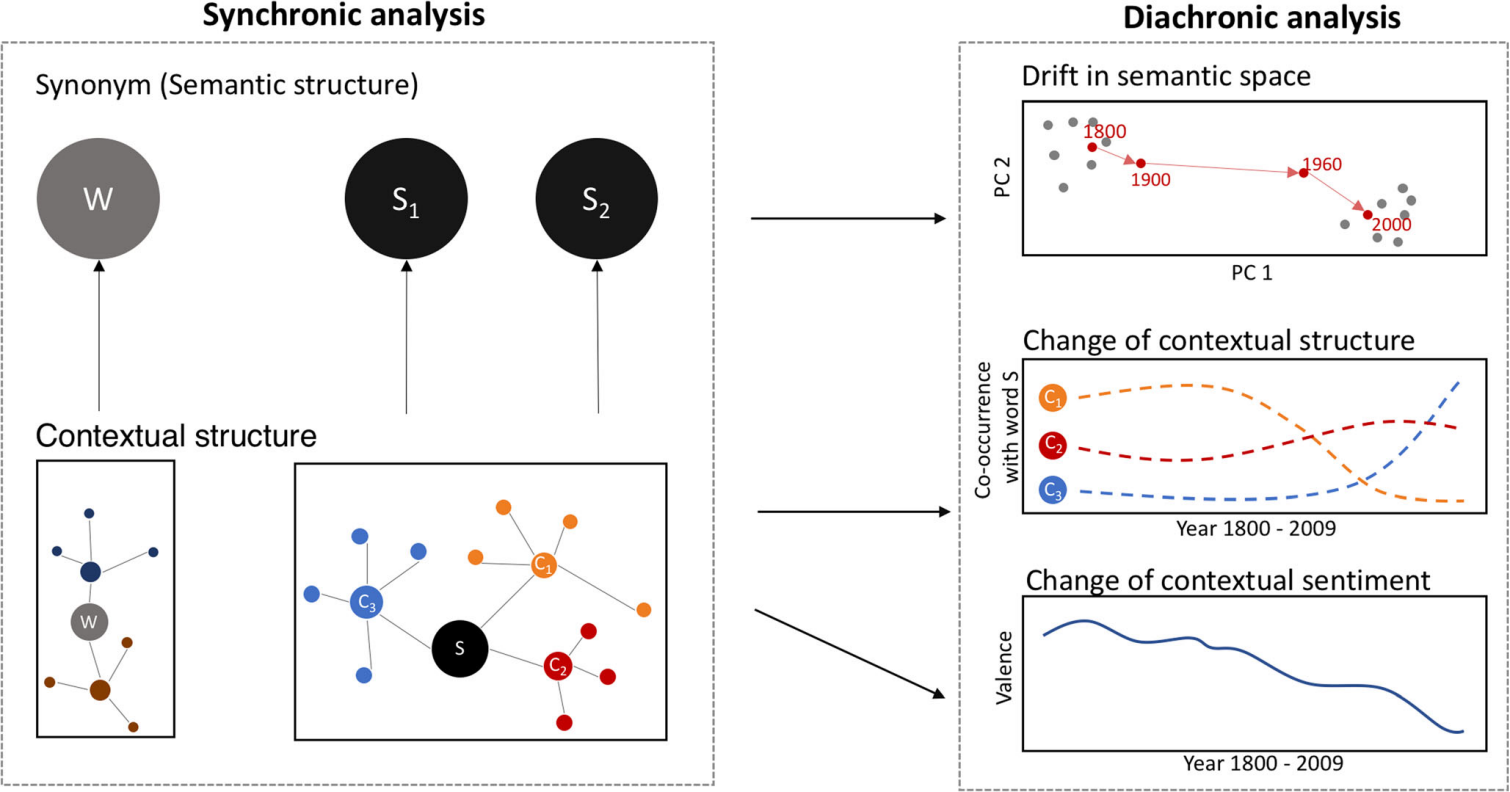
Conceptual framework summarizing the key features of the Macroscope. The Macroscope permits synchronic (left) and diachronic (right) analysis of the semantic/synonym (top) and contextual/co-occurrence (bottom) structures of words

The Macroscope provides researchers with the ability to examine two distinct but related aspects of linguistic change in individual words over historical time, as shown in Fig. 2. First, diachronic word embeddings computed from the co-occurrence matrix enable us to discover words that are semantically similar to a given word for a given year (i.e., revealing the semantic or synonym structure surrounding a word). These semantically related words will be referred to as *synonyms* for the remainder of this article (top portions of Fig. 2). Second, the co-occurrence matrix provides information regarding the context of a given word in a given year. Words that co-occur with the target word will be referred to as *context words* for the remainder of this article (bottom portions of Fig. 2).

In addition to being able to “focus” the Macroscope on the semantics and contextual structure of an individual word in a particular year, the true power of the Macroscope is harnessed when the researcher “zooms” out to obtain a bird’s eye view of changes in the semantic and contextual structure of words over historical time. Below we describe how the Macroscope can be used to examine the semantic (synonym) and contextual (co-occurrence) structures of individual words for a specific year (i.e., zooming in) and over historical time (i.e., zooming out). In the analyses described below, techniques from network analysis are employed to help with the interpretation and visualization of the synonym and co-occurrence structures of words. All analyses can be easily replicated using the Macroscope, and the user can download the network graphs along with the data used to construct the graphs.

### Synchronic semantic structure of words: Historical synonyms

How do we know what a word meant in the past? Using diachronic word embeddings, the Macroscope can quantify semantic similarity by computing the cosine distance of word embeddings for any pair of words. Therefore, a word’s historical meaning can be inferred by finding its most semantically similar words in a given time period (i.e., *synonyms*).

Anxiety and depression are conceptualized as two distinct emotions by psychologists, yet often they are experienced by the general population as the same feeling (Barrett, 2017). To examine how these concepts are represented in the written language and produced and read by people who do not necessarily have a psychology background, we used the Macroscope to identify the synonyms of *anxiety*, *depression*, and *fear* using co-occurrence data from the year 2000 (see Table 1). *Anxiety* and *depression* share many synonyms that are associated with mental disorders. In contrast, *fear*, another commonly experienced negative emotion, appears to have different synonyms from *anxiety* and *depression*.

**Table 1:** Top five closest synonyms of *depression*, *anxiety*, *fear*, *disgust*, and *anger* from the year 2000, provided by the Macroscope

Depression	Anxiety, psychosis, depressive, hyperactivity, disorder
Anxiety	Depression, mood, paranoia, panic, ideation
Fear	Dread, shame, anger, remorse, despair
Disgust	Loathing, dismay, disappointment, revulsion, sadness
Anger	Resentment, bitterness, jealousy, rage, indignation

**Fig. 3:**
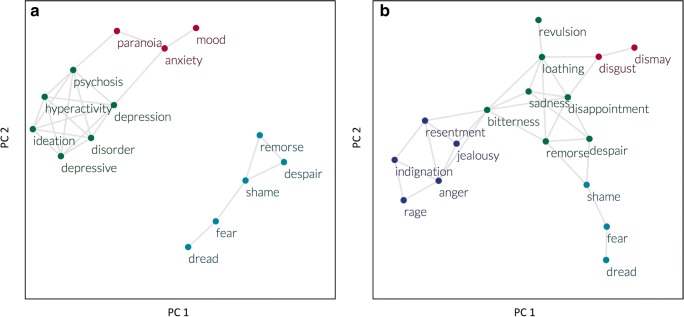
(Left) Synonym structure of *anxiety*, *depression*, and *fear*. (Right) Synonym structure of *disgust*, *fear*, and *anger*. The nodes represent the emotion concepts of interest and the top five most similar synonyms for each of the emotion concepts. The colors represent the community structure of nodes in the network, with each community represented with a different color. Community structure was detected by an algorithm proposed by Blondel, Guillaume, Lambiotte, and Lefebvre (2008)

To better capture how these three emotion concepts are related to each other, the Macroscope provides a network graph representing the semantic similarity structure of their synonyms. The nodes shown in the network represent the top five synonyms for *fear*, *depression*, and *anxiety* as identified above, as well as the words *fear*, *depression*, and *anxiety* themselves. The edges between nodes are weighted by the strength of semantic similarity between word pairs (i.e., the cosine similarity between word embeddings). Edges that are greater than a threshold of .8 are shown in the network (this value can be set by the user). If the synonyms of two words share a high degree of semantic similarity (i.e., if they are connected to each other in the semantic network), this indicates that the two words are likely to be used in similar contexts and are semantically “close” to each other. Higher semantic similarity among the synonyms of two words offers an additional layer of depth to investigate how similar are the meanings of the two words, even if the synonyms of the two words were not necessarily the same. Though previous tools have provided quantitative information about word similarity (e.g., BEAGLE from Jones & Mewhort, 2007; LSA from Landauer, Foltz, & Laham, 1998), the present example demonstrates how the Macroscope provides and visualizes additional information about the broader semantic similarity structure of words via their synonyms. Figure 3 (left panel) shows that the synonyms of *anxiety* and *depression* are synonyms of each other but are distinct from those of *fear*. Although psychologists treat anxiety and depression as two separate constructs, they appear to be used in semantically similar contexts in written language.

The same network approach used to represent concepts and their synonyms can also provide insights into the overlapping and distinctive components of two concepts. A similar analysis was conducted for the emotion words *fear*, *disgust*, and *anger*, three of the six basic emotions that are proposed to exist universally across cultures (Ekman, 1992). The results indicated that all three negative emotions intersect with some of each other’s synonyms (see Table 1). Figure 3 (right panel) shows that the concepts of *anger*, *fear*, and *disgust* share similar connections to such words as *disappointment*, *bitterness*, and *loathing*. However, each of these emotion concepts is also marked by its own unique components, which make the concepts distinct from each other: *disgust* is linked with *dismay*, *anger* with *rage and resentment*, and *fear* with *dread* and *dread*.

### Diachronic semantic structure of words: Semantic drift analysis

With diachronic language data, the Macroscope is able to track how the semantics of individual words change over time. In the following examples we show how several words “move” along a path in a semantic space defined by their historical synonyms. A longer path moving from one point in the semantic space to another indicates significant changes in a word’s semantic meaning over time. In contrast, a path that stays within a confined semantic space suggests that the word has retained its meaning over the time window examined.

Using the Macroscope, the user can conduct a semantic drift analysis by inputting the word of interest, beginning and end time points (e.g., the years 1850 and 2000), and intervening intervals (e.g., spaced every 50 years). A semantic space is then constructed for a target word by searching for its historical synonyms at the beginning time point (1850) and its modern synonyms at the end time point (2000). All synonyms’ word embeddings are taken in their modern sense (2000). The Macroscope also retrieves the historical word embeddings of the target word for each time point of interest (i.e., 1900, 1950) and aligns these historical embeddings to its modern embedding using orthogonal Procrustes (Schönemann, 1966), an algorithm to map one matrix to another of the same shape. Finally, these word embeddings are visualized in a two-dimensional space using principal component analysis (PCA). All synonyms in this two-dimensional space are represented in their modern sense. Although in reality all word meanings fluctuate over time, we elected to adopt this approach in order to provide a clearer understanding of how changes in a word’s historical meaning occur over time, as benchmarked against its modern sense.

**Fig. 4:**
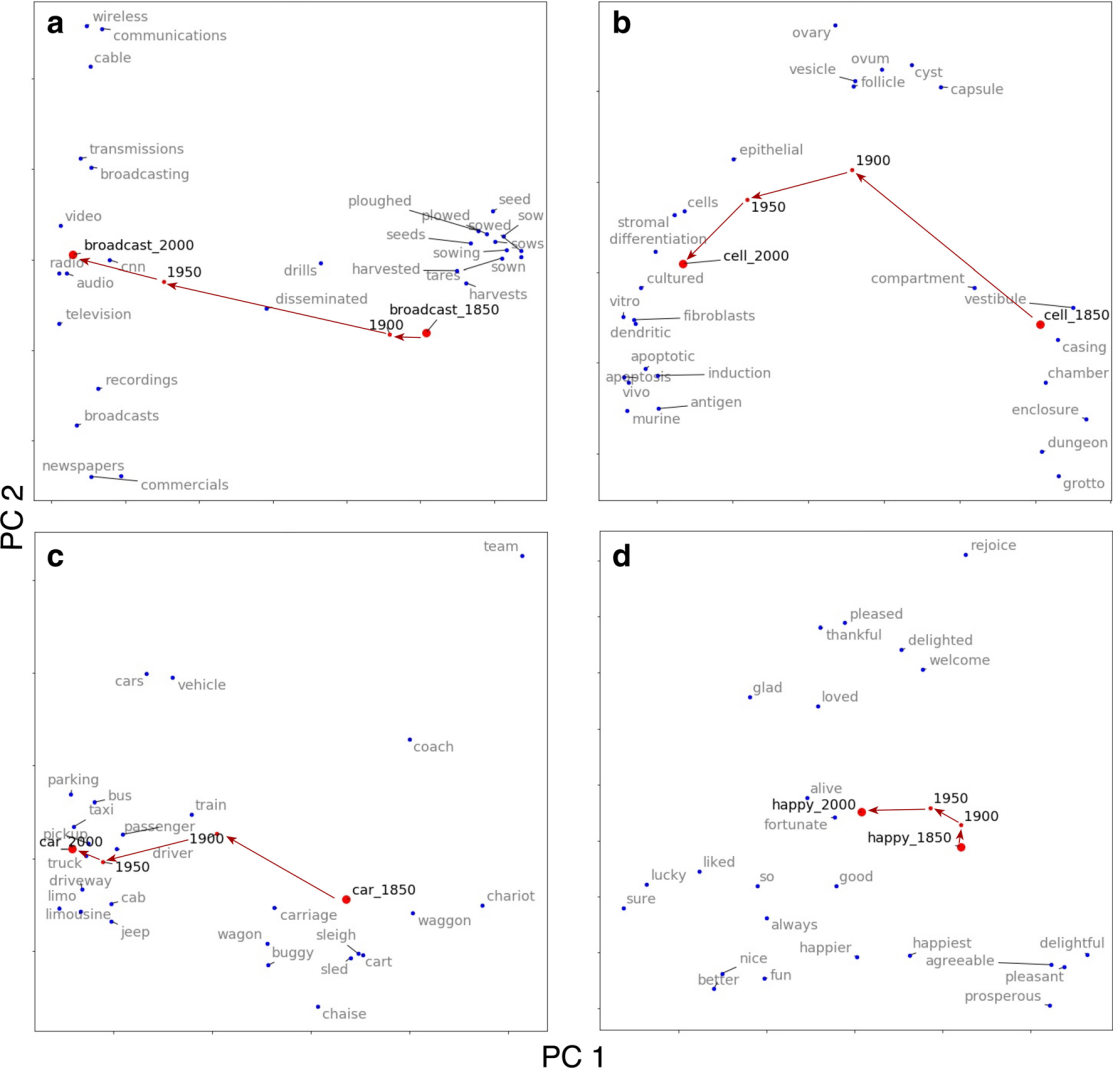
Semantic drift analysis for (top left) *broadcast*, (top right) *cell*, (bottom left) *car*, and (bottom right) *happy* from 1850 to 2000 at 50-year intervals. The blue dots indicate words that are semantically related to the target word of interest (i.e., its synonyms at the first and last time points). The path taken by the red dots indicates the “drift” in semantics of the target word from 1850 to 1900, from 1900 to 1950, and from 1950 to 2000

We used the Macroscope to examine the semantic change of three words that have been previously documented in historical linguistics (Jeffers & Lehiste, 1979). The first three panels of Fig. 4, in the top row and lower left, show semantic drift analyses of *broadcast*, *cell*, and *car* from the year 1850 to 2000 (at 50-year intervals). In 1850, the word *broadcast* referred to “disperse upon the ground by hand” and was closely associated with agricultural activity. In 2000, the word *broadcast* referred to radio and other media-related concepts. Our analysis shows that this change primarily took place between 1900 and 1950, the time period during which radio and television were invented (Fig. 4, top left). *Cell* changed its dominant meaning from “a chamber in a prison” to a biological term, and this change predominantly took place between 1850 and 1900 (Fig. 4, top right). In 1850 the word *car* referred to a horse-driven wagon, but after the automobile was invented in 1885, it quickly acquired its modern sense. The semantic drift analysis shows that by 1900 *car* was no longer associated with a wagon (Fig. 4, bottom left), but with modern transportation vehicles such as *bus* and *truck*. In addition, we conducted a similar analysis for a word that was likely to have been semantically stable over time: *happy*. The semantic drift analysis confirmed our intuitions: The word *happy* remained within the same semantic space over the past 150 years.

The semantic drift analysis shown in Fig. 4 offers a qualitative visualization of how word meanings have changed over history, but it is not easy to use such visualizations to quantitatively compare semantic stability between words (e.g., the semantic path traveled by *happy* relative to the path traveled by *broadcast* from 1850 to 2000). Previous work has examined the properties of words that appear to show the highest degree of stability over historical time (e.g., Hamilton et al., 2016; Monaghan, 2014; Pagel, Atkinson, & Meade, 2007). Since the Macroscope provides information on diachronic changes in semantics, it can be used to quantify the semantic stability of words, as is shown in Fig. 4:

$$ \mathrm{Stability}\left({w}_i,t\right)=\cos \_\mathrm{sim}\left({w}_i(T),{w}_i\left(T+t\right)\right) $$

where ${w}_i(t)$ refers to the word embedding of word *w*_*i*_ in year *t*. Semantic similarity ranges from 0 to 1. For example, the similarity of *happy* between year 1850 and 2000 is .74, much higher than the values for words that underwent greater semantic change, such as *broadcast* (.08), *cell* (.17), and *car* (.47). This allows researchers to examine potential forces that may have influenced semantic change. As a baseline for further examination, the Macroscope provides the semantic stability of a word in relation to its modern and historical word embeddings. Using this method, we retrieved the ten most stable words from 1800 to 2000. They are *and*, *the*, *when*, *his*, *he*, *they*, *him*, *in*, *them*, and *a*. A complete list of word stability between these two time points can be downloaded from the Macroscope.

### Synchronic contextual structure of words

Synonym analysis provides an accessible way to examine the semantic structure of words, based on the assumption that words that are used in similar contexts are also semantically related to each other (e.g., Jones & Mewhort, 2007). On the other hand, identifying the particular context(s) in which a word has been used can help us understand how polysemous words are used in their different senses across varying contexts, furthering our understanding of the relationship between the semantic and co-occurrence structures of words. For instance, it is possible for words to have a stable semantic/synonym structure but a varying co-occurrence structure over time. A concrete example can be seen in the word *woman*. Although the semantic meaning of the word *woman* has not changed much over the past 200 years, in recent decades the word has increasingly been used in the context of social issues surrounding feminism, gender discrimination, and abortion—contexts that were not commonly discussed during the 1800s.

The following co-occurrence networks of the words *monitor*, *option*, and *gay* show how the Macroscope can be used to understand the contextual structure of words. All networks were centered at the target word of interest. The context words, represented as nodes in the network, were selected on the basis of their PPMI value with the target word. The edges were weighted by the PPMI values between each word pair. Next, nodes with a low co-occurrence frequency with the target word and edges signaling low PPMI values were removed. During the procedure, arbitrary thresholds for parameters must be specified in order to produce meaningful network graphs. The networks presented below were constructed using a PPMI threshold of 3 and a minimum co-occurrence frequency of 200 times per ten billion words. Communities are subgroupings of nodes that are more likely to be connected to each other than to other nodes within the network. Community structures of the network are detected using an algorithm introduced by Blondel, Guillaume, Lambiotte, and Lefebvre (2008), based on modularity optimization, which uses an iterative process that defines each node as a community at the first step and merges them until modularity (a measure of the strength of the communities) is optimized.

**Fig. 5:**
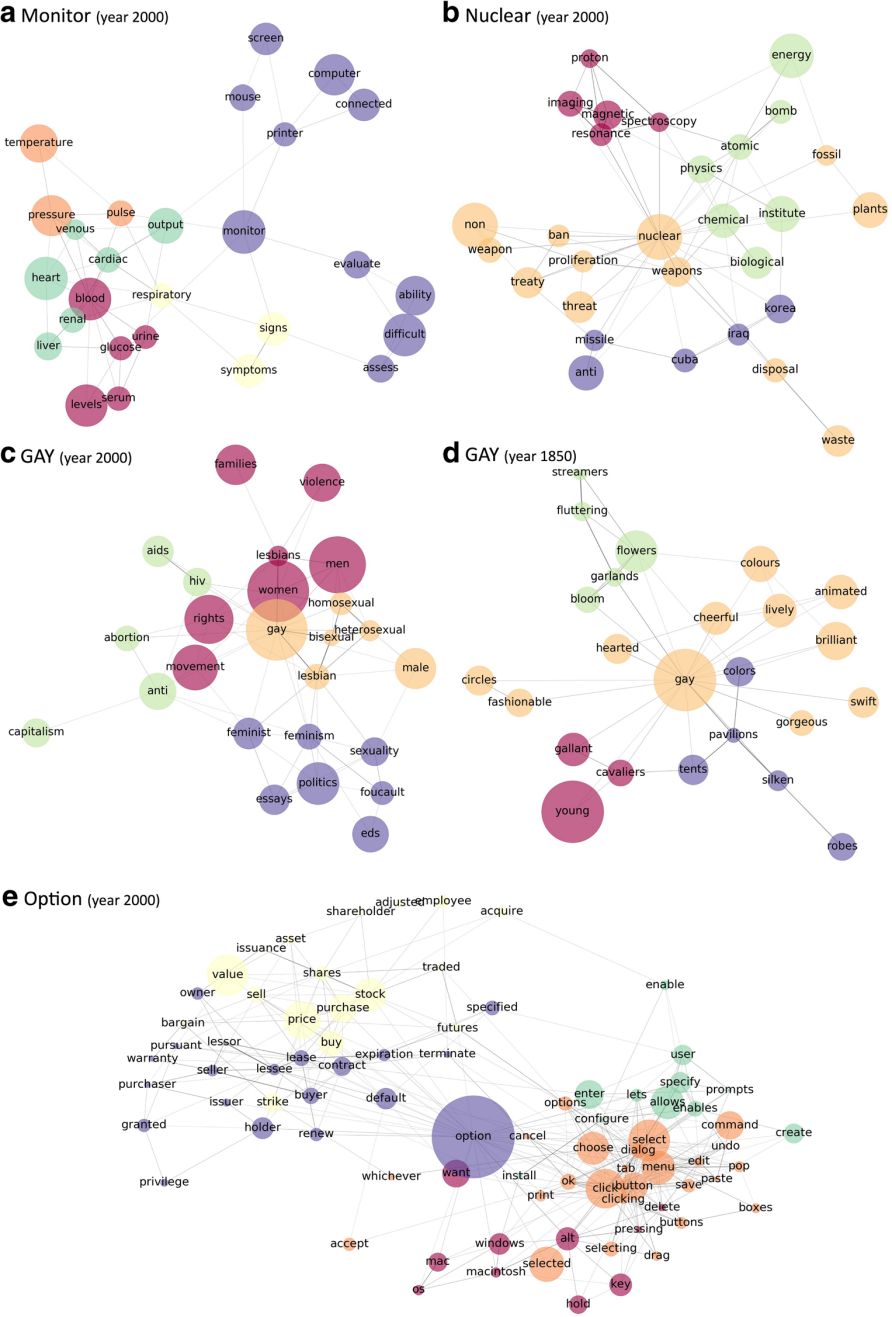
The contextual network structure of (a) *monitor*, (b) *nuclear*, (c) *gay* in year 2000, (d) *gay* in year 1850, and (e) *option*. The nodes represent the context words that co-occurred with the target word in a given year. The size of nodes is proportional to their usage frequency in a given year. The nodes were included in the networks if they had a PMI threshold greater than three with other words, and a minimum co-occurrence frequency of 200 times out of one billion words with the target word. The colors represent the community structure of nodes in the network and each community is represented with a different color

Figure 5a shows the contextual network structure of *monitor* in the year 2000. Community detection analysis of the contextual network showed approximately three distinct contexts in which the word was used: as a computer device, in healthcare-related settings, and with a group of nouns that it often accompanies. From the contextual network structure of *monitor*, one can infer that it is used as a noun or a verb. As a noun, *monitor* is often referred to as a computer device; as a verb, *monitor* is often used in medical settings.

Figure 5b shows the contextual network structure of *nuclear* in the year 2000, which shows that the word is used in a number of distinct contexts: It can refer to a power source, physical phenomena, a technology known as nuclear magnetic resonance, or a weapon associated with some countries (*Iraq*, *Cuba*, *Korea*) but not with other nuclear-armed states.

Figure 5e shows an example of what the contextual structure of a polysemous word such as *option* looks like. Other than the conventional context of choosing among various possibilities, *option* also refers to a financial instrument. As Fig. 5e shows, its contextual structure in the year 2000 was divided into two components. One involves its traditional sense, which incorporates use of the option button on a keyboard. The other component consists of finance-related terms. It is important to note that such information would not be available if one only analyzed the synonyms of *option* in the year 2000 (which are *options*, *cancel*, *default*, *item*, and *choose*), further highlighting how an analysis of a word’s contextual structure can complement the analysis of that word’s semantic structure.

As we mentioned earlier, understanding the contextual usage of a concept can be useful for inferring changes in the sociocultural environment. Figure 5c shows the context in which the word *gay* was used in the year 2000. It was not only associated with homosexuality, but also with a political movement associated with issues that extended beyond gay rights, such as feminism and abortion. Sexually transmitted diseases such as HIV and AIDS also appeared in this context, reflecting a social awareness of the association between homosexuality and the way that these diseases were transmitted among communities of gay men during the AIDS epidemic in the 1980s and 1990s. In contrast, 150 years earlier, not only did all these associations not exist, the word *gay* simply did not refer to homosexuality. The contextual structure analysis suggests that the word *gay* in 1850 was used in contexts involving fashionable clothes, cheerful mood, and pleasant colors (Fig. 5d).

### Diachronic contextual structure of words

**Fig. 6:**
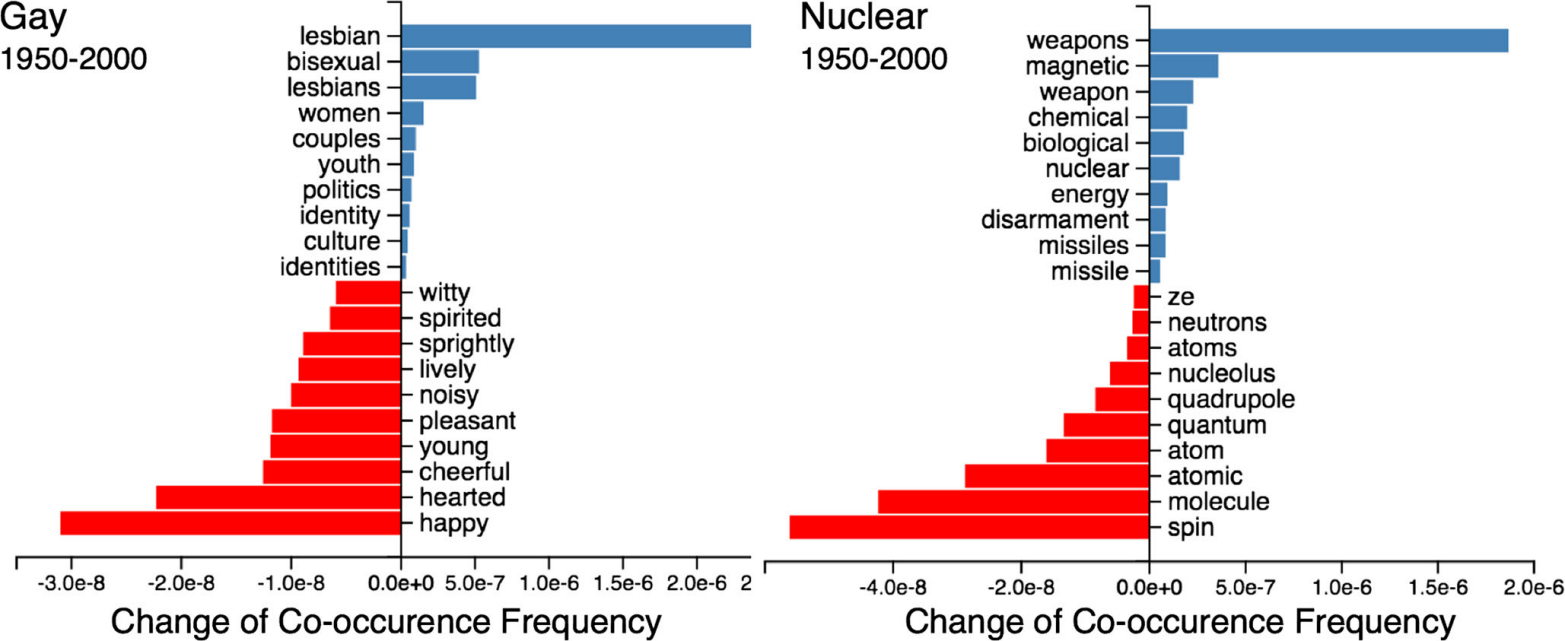
Words whose frequency of co-occurrence with *gay* and *nuclear* changed the most from 1950 to 2000. Words that increased the most in their frequency of co-occurrence with the target word from 1950 to 2000 are shown in blue near the top and words that decreased the most are shown in red near the bottom. The *x*-axes on the left and right side of the *y*-axis are scaled differently so that the *y*-axis is centered in the middle of the graph

In addition to quantifying the contextual structure of words at a static point in time, the Macroscope allows users to quantify changes in the contextual structure of words diachronically. Figure 6 shows how the frequency of co-occurrence of the words co-occurring with *gay* and *nuclear* has changed between the years 1950 and 2000. The words with the largest blue bars extending to the right (top of each *y*-axis) are those whose frequency of co-occurrence with the given word has increased the most from 1950 to 2000, whereas the words with largest red bars extending to the left (bottom of each *y*-axis) are those whose frequency of co-occurrence with the given word has declined the most from 1950 to 2000. For instance, for the word *gay*, *lesbian* and *bisexual* increased the most in their frequency of co-occurrence, whereas *happy* and *hearted* decreased the most in their frequency of co-occurrence. For the word *nuclear*, *weapons* and *magnetic* increased the most in their frequency of co-occurrence, whereas *molecule* and *spin* decreased the most in their frequency of co-occurrence, reflecting the increased usage of *nuclear* for a weapon of destruction in recent years, as compared to its scientific sense in the 1950s.

Although the previous analysis shows the largest changes in the frequency of co-occurring words between two time points, it is not completely clear to what extent a word would have “lost” its old meaning. For instance, it is possible for a word’s old meaning to still be in use, albeit not as commonly used as before. In addition, the previous analysis does not contain information regarding fine-grained changes in the frequency of co-occurring words during the time period between the two specified time points.

**Fig. 7:**
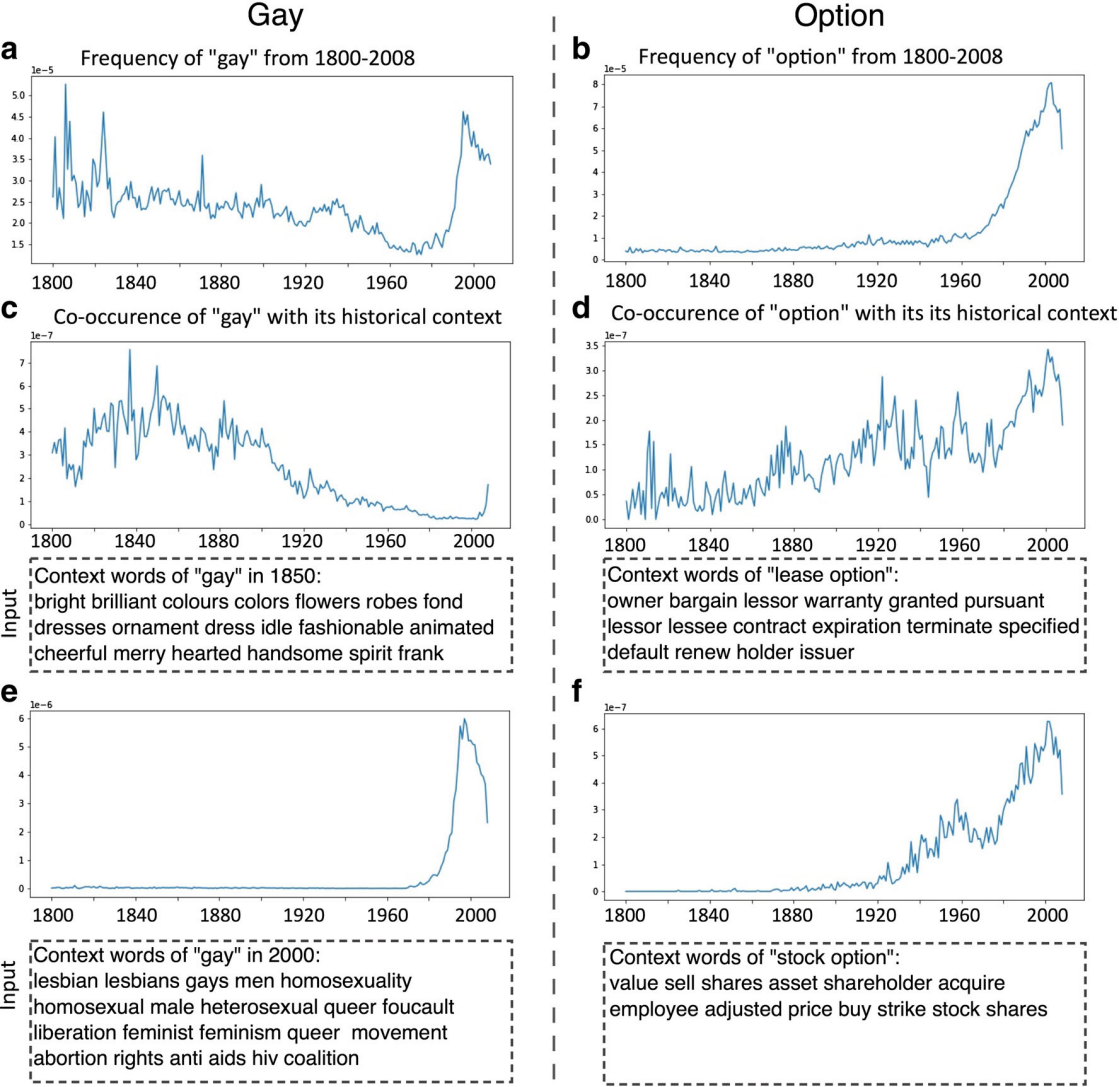
Co-occurrence frequency between the target word and its context words from 1850 and 2000. The context words were derived from the synchronic contextual structure analysis described earlier (see Fig. 5 for examples). The co-occurrence frequency was computed by summing the number of times the target word co-occurred with each single word in the list of context words

One way to address these questions would be to examine the extent to which a given word co-occurred with words found in its historical context. These context words can be obtained from the synchronic contextual structure analysis described earlier (see Fig. 5). Users of the Macroscope can also enter words that are of particular interest in their research. The co-occurrence values in Fig. 7 (on the *y*-axis) were computed by summing the number of times the target word co-occurred with each word of interest (in this case, from its historical context identified in the contextual structure analysis in Fig. 5) in each consecutive year after the historical reference year.

For instance, *gay* in 1850 co-occurred with words associated with cheerfulness, bright colors, and fashion (Fig. 5c), and in 2000 it co-occurred with words associated with homosexuality and sexually transmitted diseases (Fig. 5d). The Macroscope can take these two lists of context words and compute their respective co-occurrence frequencies with the target word *gay* in order to capture how frequently its meaning in 1850 and its meaning in 2000 have been used over the entire corpus (i.e., from 1800 to 2009). Figure 7 (left side) shows that the overall usage frequency of *gay* can largely be decomposed into two trends, with each corresponding to a different sense of *gay*. The co-occurrence between *gay* and its context words in the year 1850 declined quickly after 1900, whereas the co-occurrence between *gay* and its context words in the year 2000 emerged in the mid-1960s and increased dramatically from the 1980s. The pattern suggests that the old meaning of *gay* has been largely overwritten by its new, emerging meaning.

Another example is the word *option* (shown on the right side of Fig. 7). When looking at the contemporary contextual structure of *option* (Fig. 5e), one can easily see that the word refers to economic instruments: A *stock option* refers to stock warranted from a company to their employees as part of a remuneration package, and a *lease option* refers to a real estate contract that gives the lessor an option to buy the property. Visual inspection of Figs. 7d and f shows that a lease option probably existed in some form before the 19th century, whereas a stock option was first introduced in the 1920s, and the usage of this sense has continued to grow since the 1980s.

By combining the synchronic contextual structure analysis of words with a diachronic analysis of the co-occurrence frequencies of context words with the target word, the Macroscope provides an accessible quantitative approach to tracking the association strength between a word and its various contextual structures over history, which could be used to investigate the evolution of word meanings or cultural change over time.

### Diachronic changes in word sentiment

So far we have demonstrated how the Macroscope can be used to investigate the semantic and contextual structures of words at a specific point of time and across historical time. Below we show how the Macroscope can also be used to examine diachronic changes in word *sentiment* and how that information can be used to infer cultural changes due to urbanization and understanding the changing social perceptions of risk.

#### Example 1: Cultural changes due to urbanization

Greenfield (2013) analyzed the changing psychology of culture in the United States as a consequence of urbanization by selecting two lists of words, associated with urban and rural cultural values, respectively, and tracking their usage frequency over time. She found that words signaling urban values have proliferated in the United States over the past century, along with a declining trend among words signaling rural values. The Macroscope not only can track the usage frequencies of these words over time, but also can track the sentiment change of words over time. Here we use the Macroscope to extend Greenfield’s results by analyzing the sentiment of words that co-occurred with the words associated with urban and rural values over historical time.

**Fig. 8:**
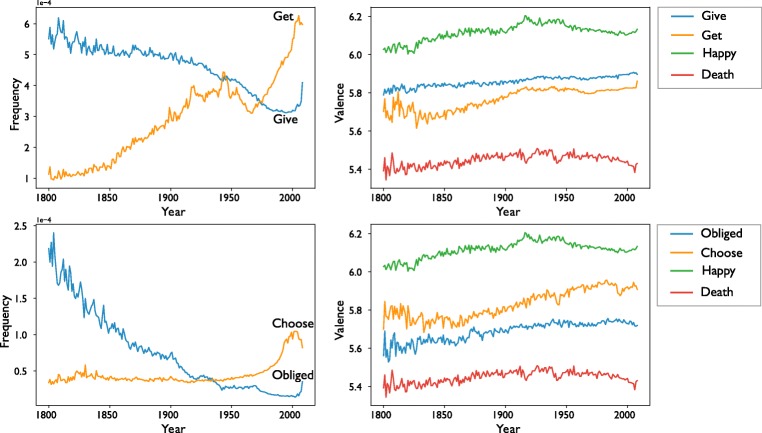
Frequency (left column) and valence (right column) from the Macroscope. The left side shows the usage frequencies for words associated with urban values (*get* and *choose* in orange) and words associated with rural values (*give* and *obliged* in blue) over historical time. The right graphs show the change in sentiment for the same words along with the change in sentiment for words such as *happy* and *death* respectively, a high- and a low-valenced word whose sentiment is stable over time

The results reproduce Greenfield’s analysis (see the left side of Fig. 8), showing that the frequency of *give* and *obliged* (rural values; in blue) decreased over time, and the frequency of *get* and *choose* (urban values; in orange) increased over time. The Macroscope adds additional information by showing that the sentiments of *get* and *choose* increased at a faster rate than did the sentiments of *give* and *obliged* (see the right side of Fig. 8). The increasingly positive sentiment of urban value words complements and extends Greenfield’s argument, because the increasing usage of words such as *get* and *choose* does not necessarily imply that urban values are viewed positively and are increasingly being adopted by people. To provide a counterexample, if a word is used more frequently but has an increasingly negative sentiment (such as the word *gay* in the 1980s during the AIDS epidemic), this concept may instead be viewed as dangerous and unfavorable.

#### Example 2: Changing social perceptions of risk

**Fig. 9:**
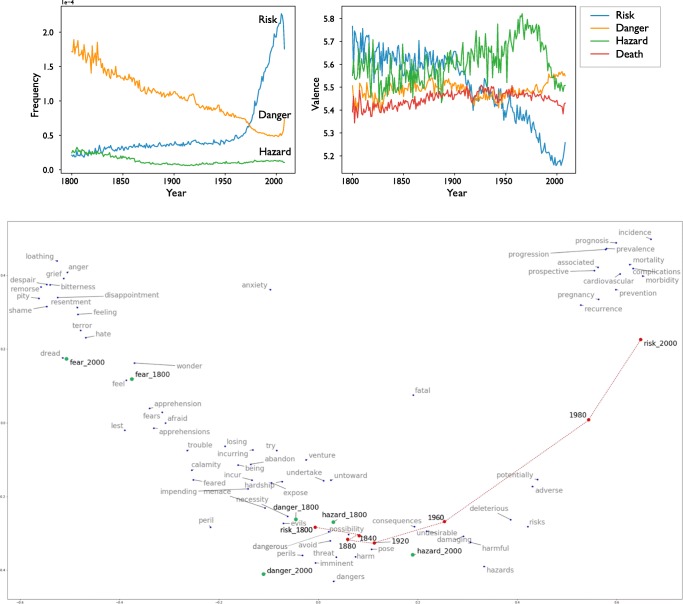
(Top left) Usage frequencies of *danger*, *hazard*, and *risk* over historical time. (Top right) Changes in the contextual sentiment of *risk*, *danger*, *hazard*, and *death* (*death* was selected as a benchmark) over historical time. (Bottom) Semantic drift of *danger*, *hazard*, and *risk* from 1800 to 2000. All figures were generated using the Macroscope

*Risk*, as defined by the Oxford English Dictionary, is a synonym for *danger*, *hazard*, and *fear*. However, sociologists and anthropologists have argued that *risk* represents more than just objective dangers or hazards in the real world. Instead, the notion of *risk* has been used to motivate social regulation and control or has acted as a surrogate for other ideological concerns (Beck, 1992). In this example, we used the Macroscope to examine the relationships between *risk* and its synonyms over the past 200 years. Our results showed that usage of *risk* experienced a rapid proliferation after the 1950s, as compared to the stable usage of *hazard* and the declining usage of *danger* (Fig. 9, top left). Correspondingly, the contextual sentiments of *danger* and *hazard* remained stable over time, whereas the sentiment of *risk* became increasingly negative (Fig. 9, top right). Output from the Macroscope (Fig. 9, bottom) shows how *risk* and its synonyms (i.e., *danger* and *hazard*) have drifted in semantic space between 1800 and 2000: *Danger* and *hazard* have had fairly limited semantic drift as compared to *risk*, which in the year 2000 was primarily associated with words related to medicine and health.

## General discussion

Language has changed over historical time, and that change is reflective of the kinds of things that people experience and believe. The goal of the present article has been to introduce the features of the Macroscope, an online algorithmic tool for zooming in and out on the semantic and contextual structure of words across historical time. The key conceptual assumptions that the Macroscope neatly capitalizes on are that words provide information about the past and that we can infer the meanings of those words through the relations they keep with other words. To summarize, the Macroscope can provide (i) synchronic and diachronic analysis of a word’s semantic structure (based on the word’s embeddings derived from the co-occurrence matrix), (ii) synchronic and diachronic analysis of a word’s contextual structure (based on word co-occurrences), and (iii) diachronic analysis of a word’s sentiment.

In the numerous examples presented above, we have provided evidence that the meanings of words can be derived through their historical context in language, which provides researchers with a new way of looking at semantic history through historical language. Importantly, these analyses can be easily conducted by anyone via the Macroscope, which can be accessed online.

The Macroscope offers numerous inroads to investigating many contemporary problems in psychology and historical linguistics (e.g., Ladd, Roberts, & Dediu, 2015). For example, what properties of words influence semantic shift (e.g., Zalizniak, 2012)? How do word senses change over time in relation to other word properties, such as frequency, concreteness, and age of acquisition (e.g., Ferrer-i-Cancho & Vitevitch, 2017; Monaghan, 2014; Zipf, 1949)? Can we use “nowcasting” methods to “backcast,” examining how word usage reflects the influence of historical events (Hills, Proto, & Sgroi, 2015; Lampos & Cristianini, 2012)? What are the additional structural properties of language that are associated with the birth and death processes of words (Pagel et al., 2007; Vejdemo & Hörberg, 2016)? To what extent have the words used in studies of age-related cognitive decline changed during the lifetime of individuals under study—for example, in studies of memory and association (Hills, Mata, Wilke, & Samanez-Larkin, 2013; Ramscar, Hendrix, Shaoul, Milin, & Baayen, 2014)? We feel that this is the tip of a large iceberg of potential questions to which the Macroscope could be applied.

Historical studies of any kind are limited in their generality by the artifacts that survive, who originally produced them, and who the artifacts were produced for. Studies of historical language are no different (see Hills & Adelman, 2015). Thus, the Macroscope is naturally limited in what it can see. As far as we know, there are no historical spoken-language corpora, which means that individuals who could not write will not be reflected (probably ever) in historical language analysis. Historical texts may have also focused on different topics over time, and therefore may not offer usage patterns that reflect common topical environments. Better understanding of these patterns and their consequences for language is part of what we hope the Macroscope can provide researchers. For example, Dubossarsky, De Deyne, and Hills (2017) showed that free association networks change nonlinearly across the lifespan, between the ages of 8 and 80. This is mostly likely due to both developmental changes associated with factors underlying human cognition and changes in the lexical environment since roughly the 1920s. Which language corpora best reflect this changing population? It is difficult to say. But studies of historical language corpora nonetheless offer inroads into understanding what language structure can explain in the absence of additional assumptions. In forthcoming iterations of the Macroscope, additional corpora will be included that will allow researchers to address specific questions about generality.

To conclude, the language people use over historical time has been a primary source of understanding people’s past beliefs and attitudes (MacWhinney, 2018). The Macroscope brings quantitative approaches to a broader range of researchers interested in understanding historical psychology through the lens of language, enabling them to test and develop hypotheses about specific patterns of word usage and semantics across history. In other words, the Macroscope is a passport to visit the foreign country of the past.

### Author note

This work was supported by a Bridges–Leverhulme Doctoral Training Centre scholarship (to L.Y.), the EPSRC (to T.E.), a National University of Singapore Overseas Postdoctoral Fellowship (to C.S.Q.S.), and a Royal Society Wolfson Research Merit Award (WM160074) and a fellowship from the Alan Turing Institute (to T.T.H.).
